# Relationship between methods of monitoring training load and physiological indicators changes during 4 weeks cross-country skiing altitude training

**DOI:** 10.1371/journal.pone.0295960

**Published:** 2023-12-15

**Authors:** Yichao Yu, Dongye Li, Yifan Lu, Jing Mi

**Affiliations:** 1 The School of Sports Coaching, Beijing Sports University, Beijing, China; 2 The School of Sports Medicine and Rehabilitation, Beijing Sports University, Beijing, China; 3 Laboratory of Sports Stress and Adaptation of General Administration of Sport, Beijing Sport University, Beijing, China; Instituto Politécnico de Santarém: Instituto Politecnico de Santarem, PORTUGAL

## Abstract

This study aimed to: (i) analyze the load characteristics of 4 weeks cross-country skiing altitude training; (ii) analyze the relationships between methods of monitoring training load and physiological indicators changes of elite male Chinese cross-country skiers during this period. Practitioners collected load data during 4 weeks of altitude training camp. Participants performed maximal oxygen uptake, lactate threshold, body composition, and skierg power test before and after the training camp to investigate the changes in physiological performance. Edwards TRIMP, Lucia TRIMP, and session rating of perceived exertion were collected as internal load. Training distance, time recorded by the Catapult module were collected as external load. The result revealed a " pyramid " pattern in the load characteristics during the altitude training camp. The correlation between luTRIMP and percent change in physiological indicators was highest. Percentage changes in lactate threshold velocity (r = .78 [95% CI -.01 to .98]), percentage changes in lactate threshold HR (r = .71 [95% CI .14- .99]), percentage changes in maximum HR (r = .83 [95% CI .19–1.00]), percentage changes in skierg power-to-weight ratio (r = .75 [95% CI -.28 to .98]) had very large relationships with luTRIMP. In cross-country skiing altitude training, training loads should be reasonably controlled to ensure that athletes do not become overly fatigued. Methods of training load monitoring that combine with athletes’ physiological characteristics and program characteristics have the highest dose-response relationships, it is an important aspect of cross-country ski training load monitoring. The luTRIMP could be a good monitoring tool in cross-country skiing altitude training.

## Introduction

Cross-country skiing is a typical endurance sports, which requires high demands on the physiological performance of athletes [1–3]. The purpose of the training is to allow athletes to develop positive physiological adaptations in response to adapted training load stimuli and improve performance levels. Physiological indicators are an objective expression of this physiological adaptation [4]. The required by modern training concepts is gradual, periodic, and specific [5], and its essence is to stimulate the athlete’s body to produce the best adaptation through the appropriate load (including volume and intensity) [6]. Practitioners need to monitor skiers’ training load and physiological stress adaptation during different training period, which allows skiers to maintain a high level of physiological performance and avoid a severe drop in performance due to overtraining [7–11].

With the popularity of various types of training monitoring equipment and the use of advanced training analysis software, it is possible to systematically analyze the training load of skiers. Training load among cross-country skiing is the cumulative stress an individual is subjected to in competition or training throughout training period [12]. According to the source of monitored indicators, there are two classification forms of load: external and internal [13]. Similar to most endurance sports, heart rate changes during cross-country skiing training are key to load monitoring [14, 15]. In the last century, Banister pioneered the Training Impulse (TRIMP) to help practitioners with load monitoring, which is a combination of training time, heart rate during training period [16]. Since then, Edwards [17] and Lucia [18] proposed two interval TRIMP calculation methods, where a linear weighting factor is used to weight the training time for the delineated heart rate intervals. On this basis, Manzi [19] have proposed TRIMP calculations based on heart rate and lactate changes. In addition to heart rate based load monitoring methods, session rating of perceived exertion (sRPE) [15, 20] and external load information collected by global positioning system (GPS) [21, 22] are also applied in ski training practice. These internal or external load-based quantification methods can help practitioners visualize the training loads to which athletes are exposed.

It is easier than ever for practitioners to access training-related load data with advances in technology. For load monitoring to be maximally effective, it is imperative that the employed methods are pertinent to significant physiological outcomes. But there is a great deal of uncertainty about the relationship between physiological performance change and these load data. Practitioners should select an appropriate load monitoring method based on the relationship between physiological performance and methods of training load, thus allowing for a more proactive approach to the development of training programmes [23]. In team sports such as soccer, some researchers have explored this phenomenon. Oliveira et al. [24] analyzed the relationship between wellness and training and match load in 13 professional male soccer players, they found that the intensity of training on the match day was correlated with indicators of sleep quality and fatigue the next day. Costa et al. [25] did not find the within-subject relationship between sleep indicators and HRV with training and match load in 20 elite female soccer players, the use of statistical methods may be a possible reason for the difference. In endurance sports, this relationship can be assessed by evaluating athletes’ physiological performance change before and after training period. A study of 8 distance runners found that athletes showed a significant increase in lactate threshold velocity after training. There were essentially relationships between improvements of lactate threshold velocity and weekly individualized TRIMP (iTRIMP). But weaker relationships between Banister TRIMP (bTRIMP) and velocity improvements at the lactate threshold [19]. Sanders et al. [26] found that a load monitoring approach incorporating the individual physiological characteristics of the athlete has the most substantial dose-response relationship in 15 well-trained cyclists. The difference of load monitoring means and the difference of sports can be one of the reasons for the different dose-response relationships.

Previous research has established a relationship between training load and physiological performance that follows a dose-response relationship. However, this relationship is complex and influenced by multiple factors, and it remains unclear whether the same holds true for cross-country ski training. This topic warrants further investigation, as cross-country skiing differs significantly from summer sports. Shedding light on this dose-response relationship can offer coaches valuable reference and data support. While a great deal of research in the past has focused on the Nordic countries, research on Chinese cross-country skiers could also enrich the training experience of emerging countries in cross-country ski training.

Therefore, the purpose of this study was to: (i) analyze the load characteristics of 4 weeks cross-country skiing altitude training; (ii) analyze the relationships between methods of monitoring training load and physiological indicators changes of elite male Chinese cross-country skiers during this period with an observation approach. Based on prior research in endurance sport, it was hypothesised that the training load characteristics conform to the classical endurance training model. Additionally, a load monitoring method that considers the physiological characteristics of the individual athlete is expected to provide the best dose-effect relationship.

## Materials and methods

### Participants and study design

**Fig 1:**
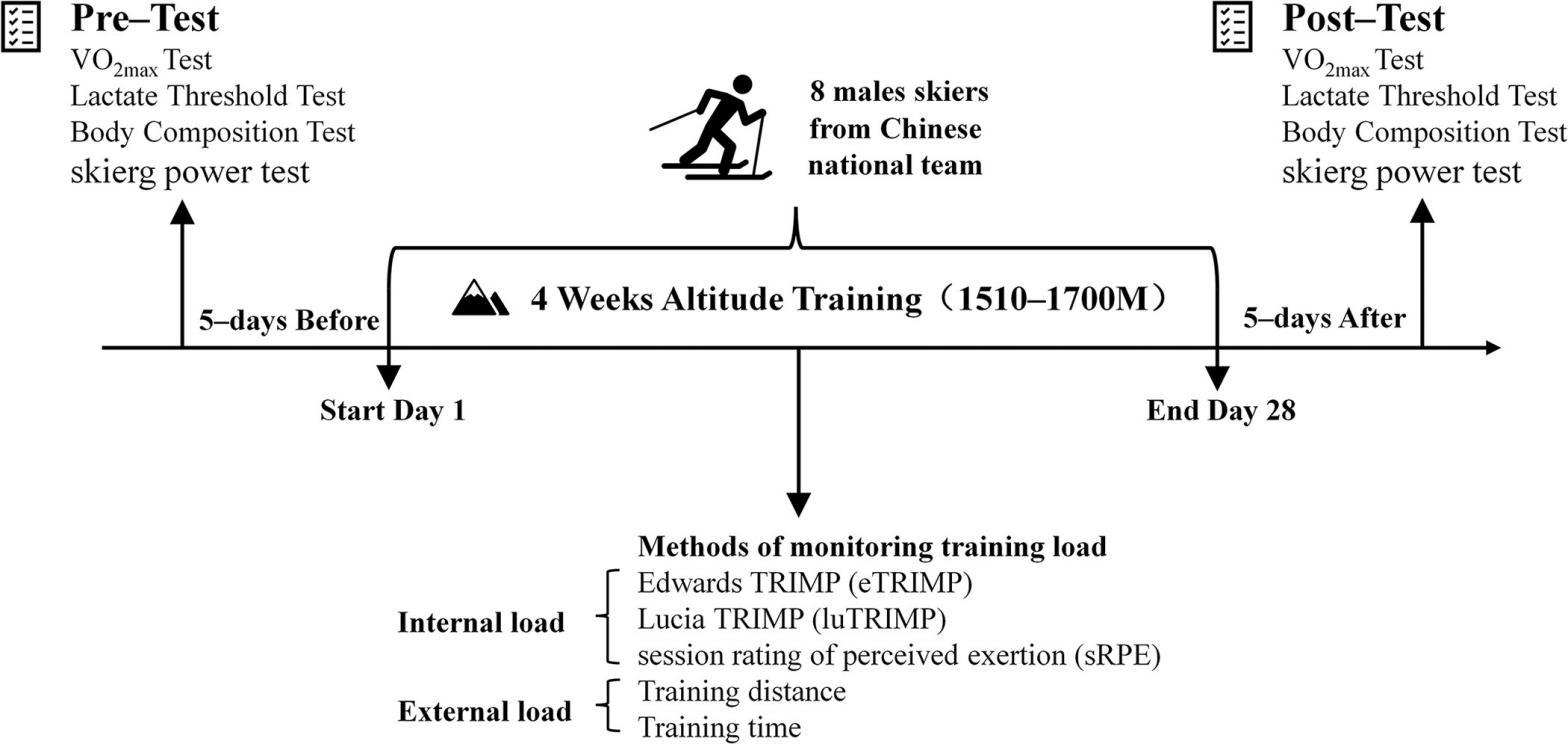
The experimental procedure of this study.

The study followed a descriptive, observational design, highlighting the relationship between methods of monitoring training load and physiological indicators changes during 4 weeks cross-country skiing altitude training (Fig 1). This study included 8 male athletes (age: 20.8±1.1 years; body mass 69.7±5.1 kg and height 179.6±5.9 cm) from the Chinese national cross-country skiing team, all of whom were elite level [27]. Although the sample size is small, it is something that often occurs in real-world studies of elite athletes, which is supported by previous studies [11, 26].

They spent 4 weeks at the Chinese national snow sports training base (sea level: 1510–1700 m, 44.5° N) from 3 May 2021 to 30 May 2021. Athletes follow a regular training plan assigned by the coaching of the national training team [28, 29], Table 1 shows the training plan example for reference.

**Table 1:** Example of a weekly training plan.

Day	Morning session	Afternoon session
Day 1	Free technique skiing 100 min: warm-up 45 min; aerobic training (15 seconds sprint + 30 seconds relax) * 5 times * 3 sets, anaerobic threshold velocity training; cool down 20 min	Running 30 min; maximal strength training: 4 exercises for upper body * 3 sets + 2 exercises for lower body * 4 sets
Day 2	Lactate Threshold Specialized Training	Free technique roller-skiing 100 min; core training 30 min; 15–20 minutes stretching
Day 3	Classical technique skiing 120 min	Running 45 min; free technique ski-specific exercises 30 min; specialized strength training 15 min; swimming 30 min
Day 4	Free technique roller-skiing 110 min: warm-up 30 min; uphill 10 sets, relax for 3 minutes between sets; cool down 20 min	Classical technique roller-skiing 80 min; core training 20 min
Day 5	Classical technique roller-skiing 150 min	Running 30 min; slow strength 3 exercises for upper body * 5 sets, 2 exercises for lower body * 5 sets, 45s of exercise between sets, 30s intervals
Day 6	Free technique skiing interval Training: warm-up 25 min; 5km * 2 sets, 3–4 min relaxed skating between sets; 10 minutes skating * 2–3 sets, 3–4 minutes relaxed skating between sets; cool down 20 min	Running 25 min; circuit strength training, 8 exercises * 2 sets, 30 seconds exercise/30 seconds rest
Day 7	Relax	Relax

During this period the athletes’ training and life were systematically monitored for training load. The daily training load data was collated by the scientific staff filled in and summarized according to the relevant templates, including the collection and calculation of internal and external load data such as heart rate, sRPE, TRIMP, training time and distance. Before and after the altitude training, athletes did physiological performance test at the Beijing Erqi National Ice and Snow Research and Training Base (55m above sea level). The tests include maximal oxygen up-take tests, lactate threshold tests, body composition tests and skierg power tests. Athletes were examined by national team doctors to ensure that there were no injuries or illnesses and were in good physical condition. All participants freely completed an informed consent form before to this investigation, expressing their desire to voluntarily engage in this study.

### Physiological performance test

In this study the lactate threshold test was completed prior to the maximal oxygen uptake (VO_2max_) test with a 5 minutes interval [30–32]. All participants performed an adequate warm-up prior to before the test. Using the treadmill (RL2500E, Rodby, Södertalje, Sweden) to evaluate the lactate threshold. The treadmill started at 7 km/h, the inclination angle was 10.5% and constant throughout, the speed increased by 1km/h every 5 minutes and the athlete has a 30 second rest period before each acceleration [30–32]. The average heart rate for the last 30 seconds of each phase was the athlete’s heart rate for that level. The athlete’s lactate is collected immediately at each step of the test and measured using a benchtop lactate meter (Boisen, EKF Industrial Electronics, Magdeburg, Germany). Ask athletes at the end of each step about their subjective feelings of fatigue (a 0–10 RPE scale). The lactate threshold refers to lactate level of 4 mmol L^−1^, treadmill speed at 4 mmol L^−1^ was calculated using linear interpolation [33].

VO_2max_ test by athletes assessed using a portable gas metabolometer (MetaMax 3B, Cortex, Leipzig, Germany) 5 minutes after the lactate threshold test [30–32]. The treadmill was inclined at the same angle as the lactate threshold test, with the starting speed 1km/h lower than the lactate threshold speed and the speed increased by 1km/h per minute until the athlete was exhausted. During the maximal oxygen uptake test, the athlete continuously wore a ventilation mask to measure the athlete’s oxygen uptake. The athlete’s heart rate was record by a heart rate belt (H10, Polar, Finland), the lactate concentration was measured 1 minute after the test and the RPE (a 0–10 scale) was recorded. The athlete’s maximum oxygen uptake (the average of the two highest and consecutive 30 s measurements), maximum HR (the highest 5s heart rate measurement), final treadmill speed and respiratory exchange ratio (RER) were recorded.

Athletes did body composition test on the morning of the test day, which used a dualenergy X-ray bone density analyzer (Luna iDXA, General Electric Company, Schenectady, NY, USA) to analyze the muscle mass of the athletes’ upper body.

Athletes performed a 30s power test using a ski ergometer (SKIERG, CONCEPT2 Inc., USA). The test was preceded by a 25-minute jogging warm-up led by a fitness coach, a 60m x 5 sets of acceleration runs, and a 1 minute x 3 sets of 75% maximum intensity warm-up using a ski ergometer. The skier was set to gear seven and the athletes performed the 30s ski ergometer test to the best of their ability. A researcher recorded the athlete’s average power output over 30 seconds and collated the records, the final result is a power to weight ratio.

### Methods of training load monitoring

All training load data of the athletes were counted and recorded by the scientific staff accompanying the team. External load such as training distance, time and speed was obtained and recorded by the Catapult module (Catapult Sports, Melbourne, Australia). The module has a GPS sensor, which can be connected to the GPS satellite system, with a sampling rate was 10Hz; it can be used for more than 5 hours for continuous training and competition monitoring [34]. Each athlete wore a special undershirt that places the module in the middle of the shoulder blades. The module was turned on 10 minutes before training to ensure good signal reception. The same module was used by every athlete during the training camp for standardization. The data were collected and analyzed after each training session by using the corporate software (Catapult openfield, Melbourne, Australia).

Athletes wore heart rate bands and watches to monitor heart rate load-related data during daily training. In this study the athlete’s maximal heart rate and lactate threshold were obtained through physiological testing, therefore two methods of TRIMP calculation based on different heart rate zones standard were considered.

The first TRIMP calculation method in this study is the approach eTRIMP proposed by Edward (Eq 1) [17].


eTRIMP=1×T1+2×T2+3×T3+4×T4+5×T5 (1)

T1: duration time when 50%HRmax <HR <60%HRmax; T2: duration time when 60%HRmax <HR <70%HRmax; T3: duration time when 70%HRmax <HR <80%HRmax; T4: duration time when 80%HRmax <HR <90%HRmax; T5: duration time when 90%HRmax <HR <100%HRmax.

The second TRIMP calculation method is the way luTRIMP proposed by Lucia [18], as shown in Eq 2.


luTRIMP=1×T1+2×T2+3×T3 (2)

T1: duration time when <HR_1_; T2: duration time when HR_1_ <HR <HR_2_; T3: duration time when >HR_2_. The HR_1_ corresponding to heart rate when blood lactate is 2 mmol L^−1^; the HR_2_ corresponding to heart rate when blood lactate is 4 mmol L^−1^.

All participants were asked for RPE 30 minutes after the training, which were tallied using a 0–10 subscale and recorded, as shown in Eq 3 [7, 20].


sRPE=RPE×durationtime(min) (3)

The rate of the physiological indicators change was calculated by Eq 4.


$$Physiological\;Indicators\;Changes\;in\;Percent\;(\%\Delta )=\frac{PostTest-PreTest}{PreTest}\times 100\%$$ (4)

### Statistical analyses

Excel 2019 and IBM SPSS 25.0 software were used to process data. All data were tested for normality using the Shapiro-Wilk test before processing, and all data were presented as mean ± standard deviation (SD). Using the paired sample T-test to compare differences in physiological performance change between two tests, the level of significance was set at p < 0.05 and the level of high significance was set at p < 0.01. Choen’sd formula was used to calculate the effect size (ES) of physiological indexes before and after the athlete’s special preparation period. ES values were evaluated according to the following criteria: 0 to 0.19 trivial, 0.20 to 0.59 small, 0.6 to 1.19 moderate, 1.20 to 1.99 large, ≥2.00 very large. Using Pearson correlation coefficients, the relationships between load quantifiers and physiological indicators changes were identified. The correlation coefficients’ uncertainties were given as 95% confidence intervals, with *p* < 0.05 being significant and *p* < 0.01 being a highly significant difference. The interpretation of the strength of the correlation coefficients is based on Hopkins: 0 to 0.09trivial, 0.1 to 0.29 small, 0.3 to 0.49 moderate, 0.50 to 0.69 large, 0.70 to 0.89 very large, 0.90 to 0.99 nearly perfect, 1.00 perfect.

## Results

**Fig 2:**
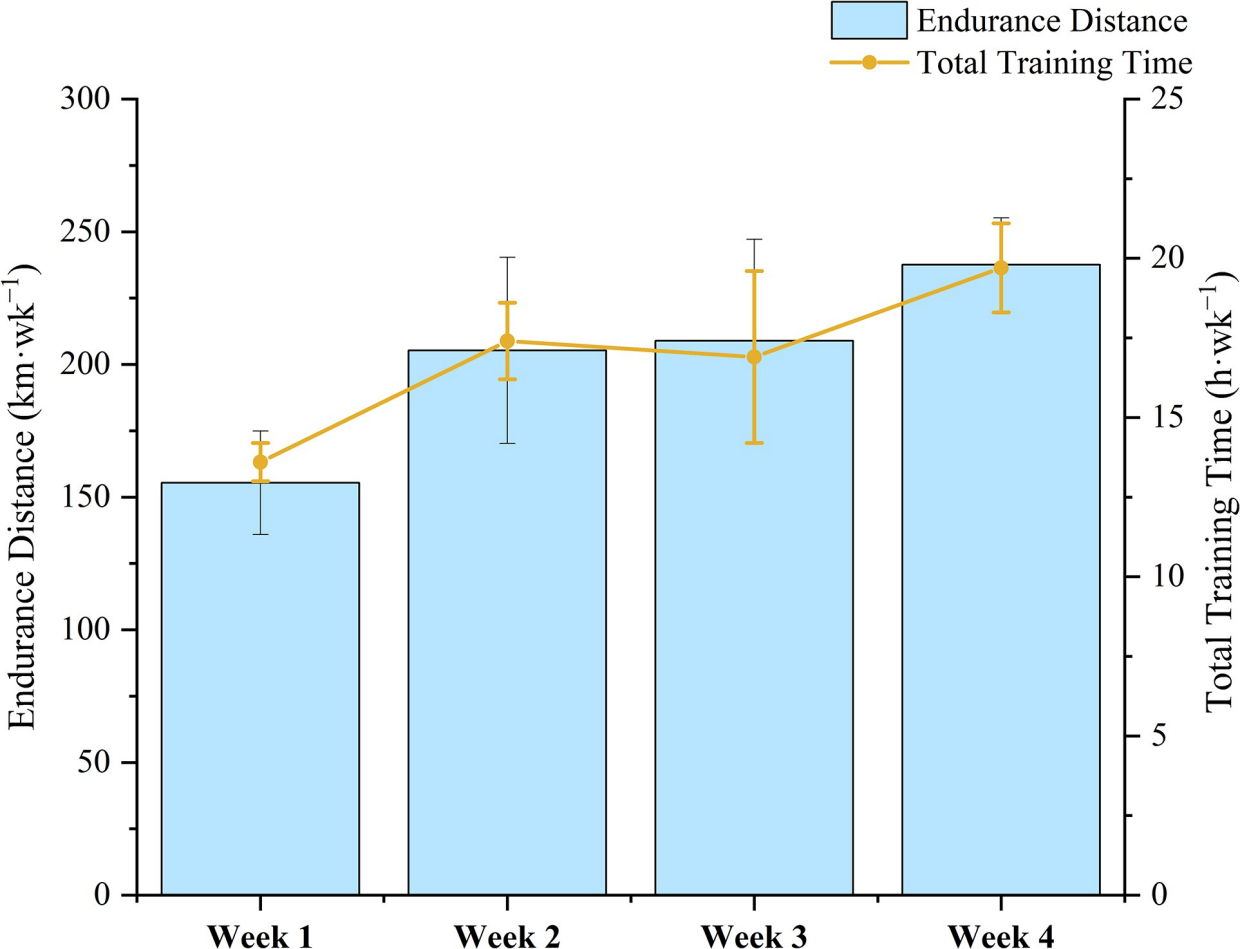
Endurance distance and total training time during training camp.

**Fig 3:**
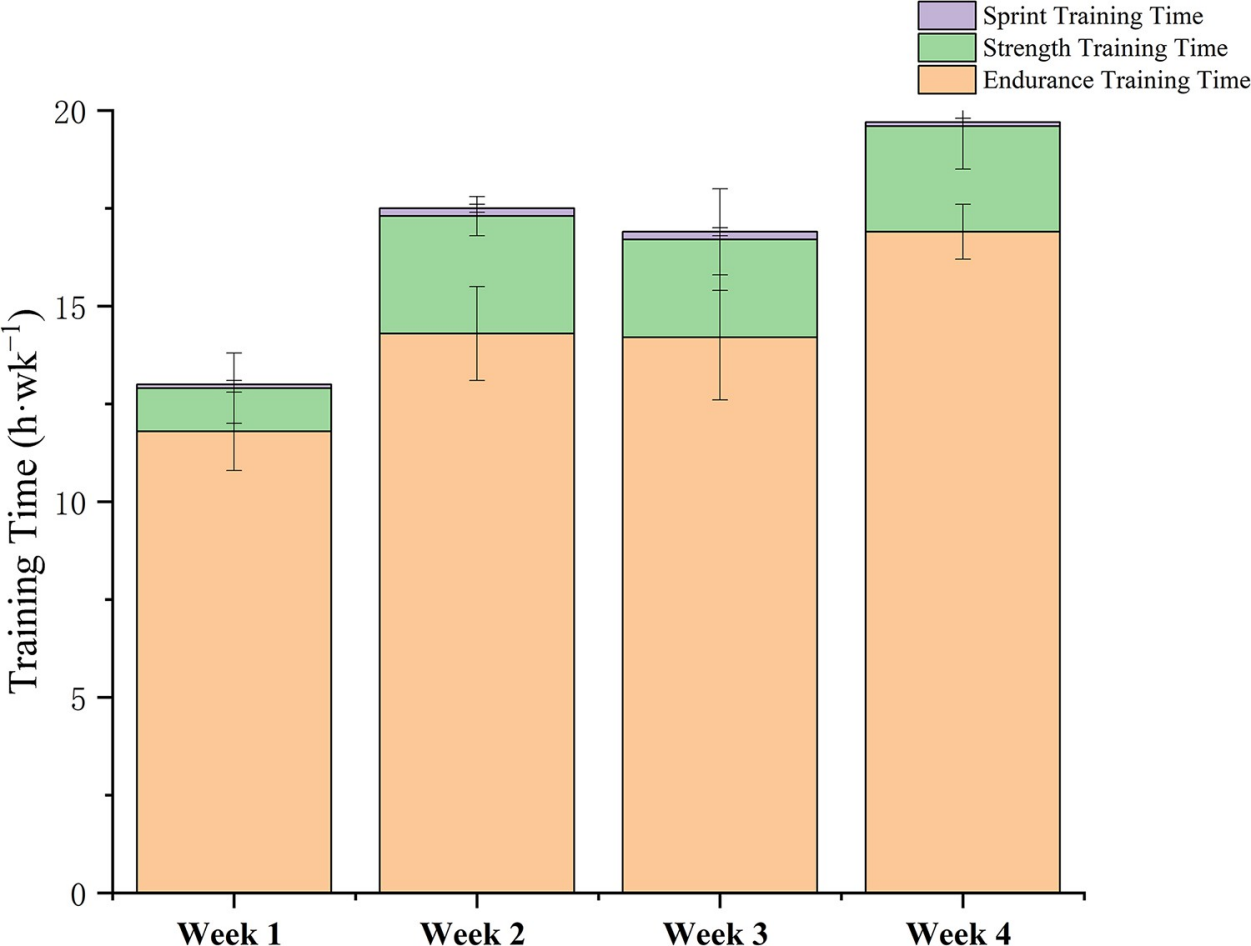
Training distribution during training camp.

**Fig 4:**
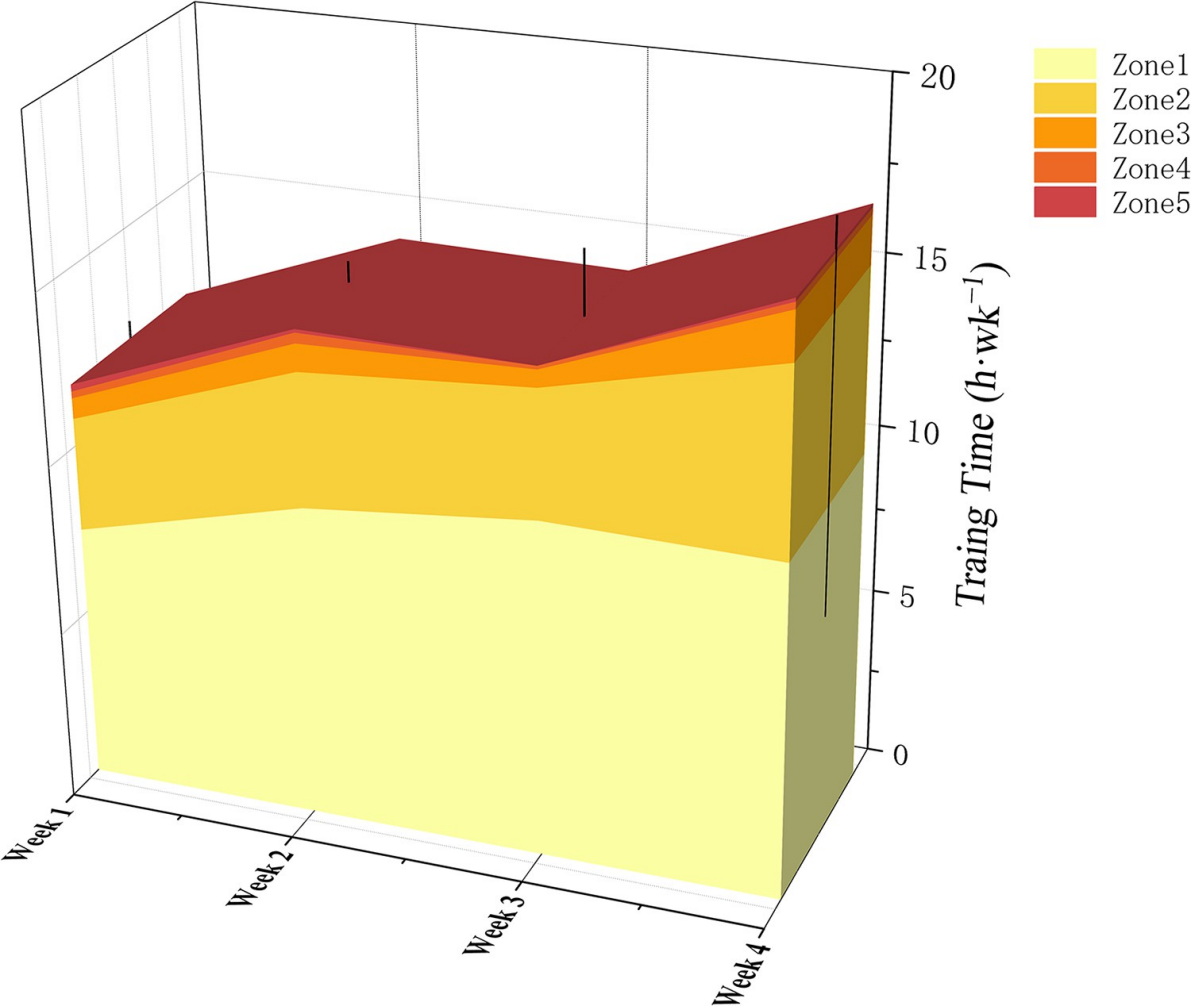
Training intensity during training camp.

The statistical results of the load characteristics of 8 Chinese male cross-country skiers during training camp are shown in and Figs 2–4. The average weekly endurance training of the 8 elite Chinese male cross-country skiers was 206.5km, the average weekly training time was 859.7 minutes, the average weekly luTRIMP value was 941.6 AU, the average weekly eTRIMP value was 1265.7 AU and the average weekly sRPE was 6953.3 AU.

As shown in Table 2, the lactate threshold velocity and heart rate increased after training (*p* < 0.05). The RER reduced after training camp (*p* < 0.05). The upper body muscle mass significant increased (*p* < 0.01) after the training camp. The skierg power-to-weight ratio increased 0.28 watt·kg^-1^ after altitude training (*p* < 0.05).

**Table 2:** Physiological performance test result.

	Pre-Test	Post-Test	Mean Difference[95%Cl]	ES
Lat velocity(m·s^−1^)	3.02 ± 0.18	3.27 ± 0.24*	0.24 [0.05–0.44]	1.18
Lat HR (beats·min^−1^)	179.5 ± 9.8	188.5 ± 9.2**	9.00 [5.08–12.92]	0.95
VO_2max_ (mL·min^−1^·kg^−1^)	73.74 ± 3.63	71.12 ± 3.14	-2.61 [-6.12 to 0.90]	0.77
VO_2max_ (L·min^−1)^	4.82 ± 0.36	4.64 ± 0.43	-0.17 [-0.33 to 0.01]	0.45
Maximum HR in VO_2max_ Test (beats·min^−1^)	197.4 ± 11.6	198.9 ± 9.1	1.50 [-3.72 to 6.72]	0.14
RER	1.23 ± 0.09	1.11 ± 0.03**	-0.12 [-0.23 to -0.01]	1.79
Maximum La in VO_2max_ Test(mmol·L^−1^)	13.31 ± 1.42	11.33 ± 2.79	-1.99 [-4.49 to 0.52]	0.89
Muscle mass whole body(kg)	55.66 ± 4.01	56.01 ± 4.02	0.36 [-0.04 to 0.76]	0.09
Muscle mass upper body(kg)	6.54 ± 0.42	6.74 ± 0.36**	0.20 [0.09–0.31]	0.51
Muscle mass trunk(kg)	27.20 ± 2.18	27.47 ± 2.00	0.27 [-0.62 to 1.16]	0.13
Muscle mass lower body(kg)	18.60 ± 1.82	18.57 ± 1.68	-0.03 [-7.34 to 0.68]	0.02
Skierg power-to-weight ratio(watt·kg^-1^)	4.38 ± 0.18	4.66 ± 0.16*	0.28 [-0.08 to 0.47]	1.64

* indicates a significant difference compared to pre-test (

* p < 0.05

** p < 0.01)

Abbreviations: Lat = lactate threshold, VO2max = maximal oxygen uptake, HR = heart rate, RER = respiratory exchange ratio.

**Fig 5:**
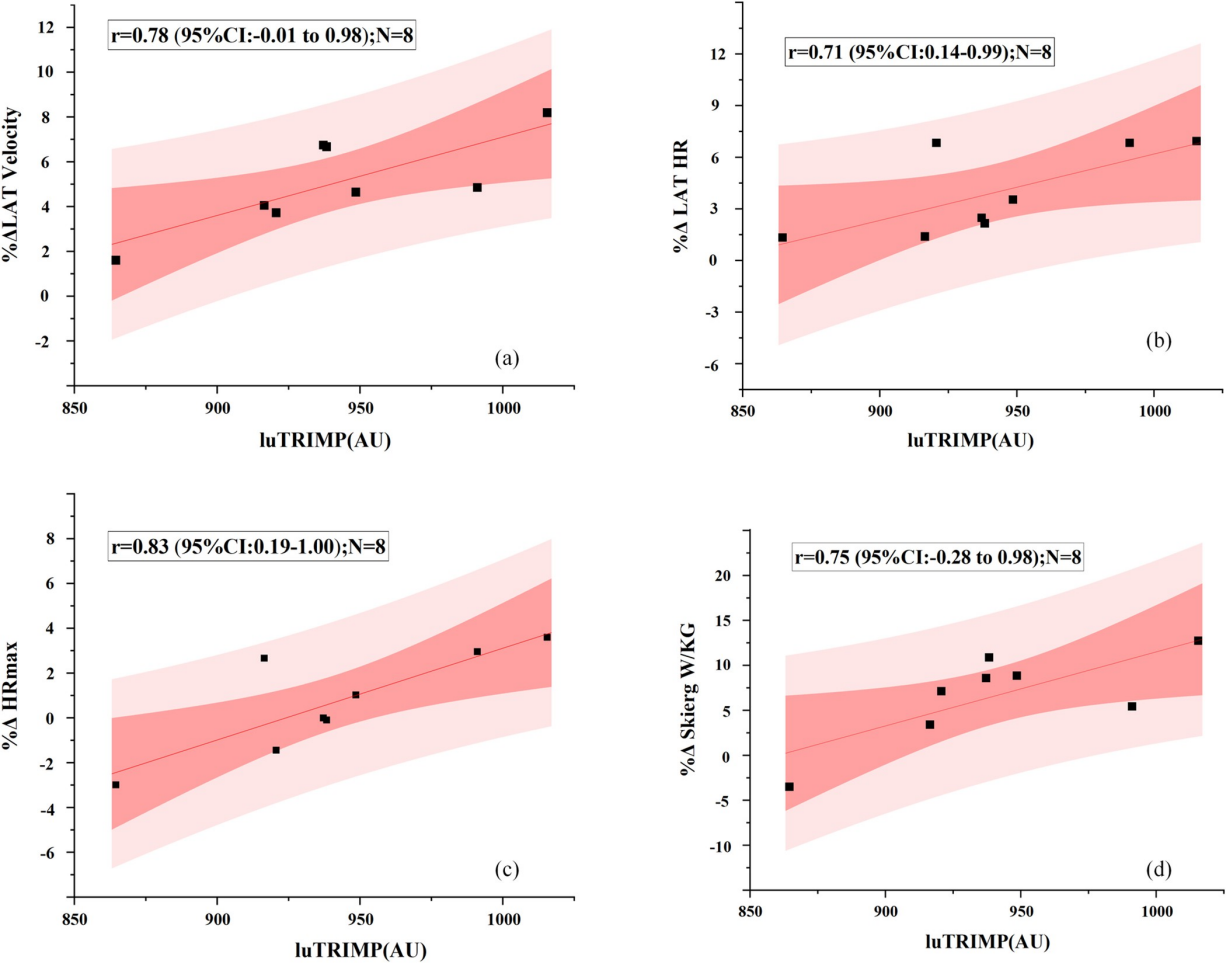
Relationship Between Average Weekly luTRIMP and Physiological Indicators Changes in Percent-age(N = 8), (a) %Δ Lat Velocity, (b) %Δ Lat HR, (c) %Δ HRmax, (d) %Δ Skierg W/Kg. Abbreviations: %Δ Lat Velocity = percentage changes in lactate threshold velocity, %Δ Lat HR = percentage changes in lactate threshold HR, %Δ HRmax = percentage changes in maxi-mum HR, %Δ Skierg W/Kg = percentage changes in skierg power-to-weight ratio, luTRIMP = Lucia training impulse.

Table 3 and Fig 5 show relationships between the methods of training load and physiological indicators changes in percentage. The correlation between average weekly luTRIMP and percent change in physiological performance was highest. Percentage changes in lactate threshold velocity (r = .78 [95% CI -.01 to .98]), percentage changes in lactate threshold HR (r = .71 [95% CI 0.14–0.99]), percentage changes in maximum HR (r = .83 [95% CI .19–1.00]), percentage changes in skierg power-to-weight ratio (r = .75 [95% CI -.28 to .98]) had very large relationships with average weekly luTRIMP. There were very large relationships between average weekly eTRIMP and maximum HR changes in percentage. Percentage changes in skierg power-to-weight ratio were very largely related to average weekly sRPE. There were very large relationships between Total Distance and percentage changes in maximum HR. The correlation between Total Time and percent change in upperbody muscle mass was very large.

**Table 3:** Relationship between methods of training load quantification and physiological indicators changes in percentage.

	Total Distance	Total Time	luTRIMP	eTRIMP	sRPE
%Δ Lat Velocity	.49[-.51 to .39]	.60[-.13 to .93]	.78*[-.01 to .98]	.42[-.52 to .86]	.62[-.31 to .94]
%Δ Lat HR	.44[-.37 to .92]	.49[-.28 to .97]	.71*[0.14–0.99]	.38[-.39 to .91]	.52[-.12 to .92]
%Δ VO_2max_Rl	.68[.31-.95]	.39[-.52 to .97]	.59[-.19 to .96]	.61[-.06 to .99]	.50[.01-.92]
%Δ VO_2max_Ab	.69[.33-.97]	.42[-.54 to .93]	.63[-.26 to .91]	.57[-.14 to .98]	.61[.21-.94]
%Δ HRmax	.81*[.36-.96]	.61[-.20 to .98]	.83*[.19–1.00]	.79*[.29-.97]	.66[-.24 to .97]
%Δ Skierg W/Kg	.62[-.34 to .94]	.60[-.10 to .92]	.75*[-.28 to .98]	.50[-.28 to .86]	.72*[-.41 to .96]
%Δ Upperbody Muscle Mass	.54[-.13 to .96]	.78*[.44-.99]	.62[-.11 to .94]	.65[-.02 to .95]	.53[-.34 to .97]

* Indicates a significant at the 0.05 level (2-tailed)

** indicates significant at the 0.01 level (2-tailed). Abbreviations: %Δ Lat Velocity = percentage changes in lactate threshold velocity, %Δ Lat HR = percentage changes in lactate threshold HR, %Δ VO_2max_Rl = percentage changes in maximal oxygen uptake relative values, %Δ VO_2max_Ab = percentage changes in maximal oxygen uptake absolute values, %Δ HRmax = percentage changes in maximum HR, %Δ Skierg W/Kg = percentage changes in skierg power-to-weight ratio, %Δ Upperbody Muscle Mass = percentage changes in upperbody Muscle Mass, sRPE = session rating of perceived exertion, eTRIMP = Edwards training impulse, luTRIMP = Lucia training impulse.

## Discussion

The purpose of this study were to: (i) analyze the load characteristics of 4 weeks cross-country skiing altitude training.; (ii) analyze the relationships between methods of monitoring training load and physiological indicators changes of elite Chinese cross-country skiers. The result revealed a " pyramid " pattern in the load characteristics of 8 Chinese male cross-country skiers during the altitude training cycle. The highest relationships were found in luTRIMP, the results of this study show that load monitoring methods that combine program characteristics and individual physiological characteristics have the highest relationships with physiological performance change, as opposed to internal and external load metrics that only combine average exercise intensity.

The essence of sports training is the precise control of the training load, and detailed statis-tics on the training load can help to understand the athletes’ training situation in a certain cycle and make timely adjustments accordingly [21]. The load statistics show a " pyramid " training pattern in terms of overall training load and intensity during this period. Compared to similar training cycles for world elite cross-country skiers (polarised training pattern), endurance training time is relatively low at 1.4h (approximately 15.7h for the international elite level), strength training time is 1.6h higher per week (approximately 0.7h for the international elite level) and speed training time is similar. In terms of training load intensity, the average weekly LIT is 1.2h lower than that of international elite athletes (about 14.4h), the MIT is about 0.3h higher per week (about 0.5h for international elite level) and the HIT is about 0.5h lower per week (about 0.8h for international elite level). The reasons for the discrepancy with elite international athletes may be closely related to the short years of ski-specific training. Due to their short training period, athletes have not yet developed good glide economy and are prone to overrange glide heart rates during the long, low-intensity aerobic glides required by the training plan [2, 3]. The long duration of strength training is due to the fact that the national cross-country skiing coach, in response to the lack of specific strength in our athletes, has designed special strength training after long periods of low intensity aerobic training to help the athletes to achieve the appropriate strength requirements as much as possible.

Scientific assessment of the relationship between external load, internal load and sports performance can help coaches and sports researchers to better examine the dose-response relationships resulting from sports training and to make a meaningful impact on training [10]. The strongest dose-response relationship between luTRIMP and physiological performance was found in this study, where it was shown that internal loads in cross-country skiers were closely related to maximal oxygen uptake and lactate threshold heart rate, and that changes in these in-ternal load monitoring methods were correlated to a moderate to high degree with changes in physiological performance [35]. Consistent results were also seen in the hurling [36] and soccer players [37]. The correlation between eTRIMP and physiological indicators changes in this study was much less than luTRIMP. The biggest reason is that eTRIMP was obtained based on data analysis of a certain group of athletes and not based on individual physiological performance tests, and the division of heart rate intervals deviated from the sports characteristics of cross-country skiing [26]. Some researchers have pointed out the limitations of load monitoring tools that do not incorporate the physiological characteristic points of the sport, as the intensity of an athlete’s training can change depending on the environment, weather and tactics. In an experiment with cyclists, researchers found a weak dose-response relationship between bTRIMP and exercise performance based solely on the generalized exercise blood lactate equation [38].

sRPE as a load monitoring indicator has been widely used in the field of sports training [20, 39]. However, the dose-response relationship between sRPE and physiological indicators changes in this study was still lower than that between heart rate load-related metrics, which is consistent with the better relationship between heart rate based load monitoring methods and oxygen consumption than RPE based methods derived by Wallace et al. [40]. It should also be considered that the short-term training intervention in this study and the relatively high altitude may have had an additional effect [41, 42]. It should also be considered that cross-country skiing differs from other sport groups in that the training pattern of prolonged exposure to cold temperatures may lead to a bias in the athletes’ subjective perception of fatigue, which in turn may lead to a bias in the RPE values compared to laboratory tests, resulting in an error in the sRPE monitoring of training load [43]. It should also be noted that load monitoring in this study was limited to the training period, with evidence suggesting that sRPE monitoring values during competition better reflect an athlete’s true internal load level. Furthermore, a significant difference was found in the psychological state of athletes between training and competition, with the former being more monotonous and the latter generating stronger emotional responses, leading to notable psychological differences. Differences in training and competition intensity can result in varying effects of load quantification [44].

The correlation between load monitoring methods and changes in physiological performance in this study, whilst moderate, is far less than has been found in practice in other projects. Temperatures, wind speeds, snow quality and snow waxes in alpine environments differ from those in plains, and the accumulation of fatigue due to low oxygen exposure may lead to biased results. Differences in the duration of physiological performance tests may also contribute to this result, with some studies showing a stronger effect relationship between longer duration physiological tests and load quantifiers [9]. Data collection from sports training practice is more difficult to control than laboratory research design, which inevitably leads to variability in the data collected [45]. Considering the above factors, the relationships in this study should be interpreted with caution. Therefore, in future studies that wish to apply load monitoring methods to measure or analyze the dose effects of physiological performance in cross-country skiers in a given cycle, more consideration should be given to the influence of complex confounding factors on the results [46].

### Limitations

As an observational study, this study still has a few flaws in it. Firstly, the sample size in this study was relatively small due to design limitations, which is usually the norm in real-world sports science research of elite athletes. Additionally, the training period only lasted for 4 weeks, which could limit the study’s ability to fully capture the physiological adaptations to altitude training, as well as the acute and chronic effects of applied training. Finally, the load monitoring methods used in this study were very limited due to practical constraints and more different load monitoring methods should be considered for future analysis. Therefore, future research should aim to expand the sample size and increase the number of data points to improve statistical analysis. Linear mixed models could be considered to mitigate the impact of individual differences on the outcomes [25]. It is important to note that competitive sports prioritise results, hence more attention should be given to sports performance indicators.

## Conclusion

The study revealed a " pyramid " pattern in the load characteristics of 8 Chinese male cross-country skiers during the 4 weeks altitude training short cycle in preparation for the Beijing Winter Olympics, with a lower duration of LIT and a higher duration of MIT than at the elite international level. In the process of training on the altitude, it is important to control LIT while improving the other basic skills of the athletes to avoid fatigue and other phenomena. Training load monitoring methods that combine with program characteristics and individual physiological characteristics have the highest dose-response relationships with physiological indicators changes. Indicating that this is an important factor in cross-country skiing training load monitoring, luTRIMP could be used as a good internal load monitoring tool in cross-country skiing. The use of non-invasive load monitoring methods incorporating individual physiological characteristics of athletes should be considered in future cross-country skiing training monitoring to reveal changes in athletes’ physiological performance after a particular training intervention, in order to improve cross-country skiing training monitoring and load evaluation systems.

### Practical applications

Training load monitoring can better help practitioners understand the load characteristics during a cycle, especially in specific training environments. For instance, when coaches identify that athletes are experiencing high loads based on the monitoring outcomes, they should timely modify the training programme to avoid overreaching or overtraining. It is also valuable for practitioners to strengthen the dose-respond relationship between load quantification methods and physiological indicators. This can enable the practitioners to understand the physiological adaptations that will occur in the athlete through training loads alone. Although the relationship can only be established for a specific group of athletes, providing an evidence-based framework can aid in the development of training programs. Moreover, this relationship might be impacted due to cold weather in winter sports. Practitioners ought to choose load monitoring methods that are specific to the training environment and program characteristics. Future research should consider better statistical methods based on more data to evaluate the dose-response relationship between longer training and more physiological indicators in cross-country skiing.
